# Interferon-α blocks HIV-1 infection in non-dividing myeloid cells despite SAMHD1 degradation and high deoxynucleoside triphosphates supply

**DOI:** 10.1186/1742-4690-10-S1-P69

**Published:** 2013-09-19

**Authors:** Loic Dragin, Laura Nguyen, Hichem Lahouassa, Adèle Sourisce, Baek Kim, Cecilia Ramirez, Florence Margottin-Goguet

**Affiliations:** 1 Institut Cochin, CNRS UMR81, Inserm U101604, Paris, France; 2 Université Paris Descartes, Paris, France; 3 University of Rochester Medical Center, Rochester, NY, USA

## Background

Interferon-α (IFN-α) potently inhibits both the early and late phases of HIV replication by inducing diverse unknown antiviral host factors. The dGTP-regulated deoxynucleoside triphosphate (dNTP) hydrolase SAMHD1 is a restriction factor that inhibits the reverse transcription (RT) of HIV. SAMHD1 depletes dNTP levels in quiescent cells such as myeloid cells or resting CD4+ T lymphocytes. HIV-2 and its SIVsm and SIVmac close relatives encode a protein termed Vpx that counteracts this antiviral mechanism of “nucleotide depletion” by promoting SAMHD1 degradation, thus allowing the RT of retroviruses to proceed. It is also proposed that Vpx targets the IFN-α-induced APOBEC3A (A3A) antiviral protein for degradation. Here, we investigated whether IFN-α cooperates with nucleotide depletion to counteract HIV.

## Materials and methods

Peripheral blood mononuclear cells from the blood of different anonymous donors were obtained and monocytes as well as CD4+ T cells were isolated by positive selection on magnetic microbeads (Milteny Biotec). Monocyte-derived macrophages (MDMs) and CD4+ T cells were used to study IFN-α effects on SAMHD1 expression, Vpx-induced SAMHD1 degradation, Vpx-mediated rescue of HIV-1 transduction and on the dNTP supply.

## Results

IFN-α inhibited HIV-1 transduction in monocytes and in MDMs while SAMHD1 expression was not up-regulated. Vpx triggered SAMHD1 degradation in IFN-α treated cells, and weakly restored HIV-1 transduction from the IFN-α block. Vpx helper effect towards HIV-1 transduction was gradually inhibited with increasing doses of IFN-α. dNTP levels were not significantly affected in MDMs and CD4+ primary activated T lymphocytes by IFN-α and, in correlation with SAMHD1 degradation, restoration of dNTP levels by Vpx was efficient in MDMs treated with the cytokine. In contrast, IFN-α inhibited Vpx-mediated SAMHD1 degradation in THP-1 cells, where, accordingly, Vpx could not rescue HIV-1 transduction.

## Conclusion

Our results suggest that the early antiviral effect of IFN-α results from a mechanism independent of nucleotide depletion in MDMs. In addition, they indicate that the macrophage-like THP-1 cell line may provide a system to characterize an IFN-α-induced cell response that inhibits Vpx mediated SAMHD1 degradation.

